# Guardian Reports of Children's Sub-optimal Oral Health Are Associated With Clinically Determined Early Childhood Caries, Unrestored Caries Lesions, and History of Toothaches

**DOI:** 10.3389/fpubh.2021.751733

**Published:** 2021-12-24

**Authors:** Emily P. Imes, Jeannie Ginnis, Poojan Shrestha, Miguel A. Simancas-Pallares, Kimon Divaris

**Affiliations:** ^1^Doctor of Dental Surgery (DSS) Curriculum, Adams School of Dentistry, University of North Carolina at Chapel Hill, Chapel Hill, NC, United States; ^2^Division of Pediatric and Public Health, Adams School of Dentistry, University of North Carolina at Chapel Hill, Chapel Hill, NC, United States; ^3^Department of Epidemiology, Gillings School of Global Public Health, University of North Carolina at Chapel Hill, Chapel Hill, NC, United States

**Keywords:** parents, subjective oral health, children, dental caries, pediatric dentistry

## Abstract

**Background:** Parents'/guardians' perceptions of their children's oral health are useful proxies of their clinically determined caries status and are known to influence dental care-seeking behavior. In this study, we sought to examine (1) the social and behavioral correlates of fair/poor child oral health reported by guardians and (2) quantify the association of these reports with the prevalence of early childhood caries (ECC), unrestored caries lesions and toothaches.

**Methods:** We used guardian-reported child oral health information (dichotomized as fair/poor vs. excellent/very good/good) obtained *via* a parent questionnaire that was completed for *n* = 7,965 participants (mean age = 52 months; range = 36-71 months) of a community-based, cross-sectional epidemiologic study of early childhood oral health in North Carolina between 2016 and 2019. Social, demographic, oral health-related behavioral data, and reports on children's history of toothaches (excluding teething) were collected in the same questionnaire. Unrestored ECC (i.e., caries lesions) was measured *via* clinical examinations in a subset of *n* = 6,328 children and was defined as the presence of one or more tooth surfaces with an ICDAS ≥ 3 caries lesion. Analyses relied on descriptive and bivariate methods, and multivariate modeling with average marginal effect (A.M.E.) estimation accounting for the clustered nature of the data. Estimates of association [prevalence ratios (PR) and adjusted marginal effects (AME) with 95% confidence intervals (CI)] were obtained *via* multilevel generalized linear models using Stata's *svy* function and accounting for the clustered nature of the data.

**Results:** The prevalence of fair/poor oral health in this sample was 15%–it increased monotonically with children's age, was inversely associated with parents' educational attainment, and was higher among Hispanics (21%) and African Americans (15%) compared to non-Hispanic whites (11%). Brushing less than twice a day, not having a dental home, and frequently consuming sugar-containing snacks and beverages were significantly associated with worse reports (*P* < 0.0005). Children with fair/poor reported oral health were twice as likely to have unrestored caries lesions [prevalence ratio (PR) = 2.0; 95% confidence interval (CI) = 1.8-2.1] and 3.5 times as likely to have experienced toothaches [PR = 3.5; 95% CI = 3.1-3.9] compared to those with better reported oral health.

**Conclusions:** Guardian reports of their children's oral health are valuable indicators of clinical and public health-important child oral health status. Those with fair/poor guardian-reported child oral health have distinguishing characteristics spanning socio-demographics, oral-health related practices, diet, and presence of a dental home.

## Introduction

Early childhood caries (ECC) is a world-wide clinical and public health problem; it affects an estimated 600 million children and remains largely untreated (1). Globally, the mean ECC prevalence has been estimated to be 24% for children younger than 36 months and 57% for children aged 36-71 months. Furthermore, a significant association has been shown between higher economic growth and higher ECC prevalence at the individual country level (2). Though dental caries physically impacts children with possible manifestations including pain and infection, it also has an impact on a child's quality of life. Studies have linked dental caries with school absences, poor school performance, difficulty eating, trouble sleeping, and difficulty paying attention in class (3, 4).

Remarkably, despite its recognized multilevel consequences (5) and major advances in the science and practice of dentistry, the prevalence of ECC has not followed the declines observed among adults. In fact, besides being on the increase in some parts of the world, ECC is characterized by marked disparities, with children in families from socially disadvantaged or racial/ethnic minority backgrounds experiencing a disproportionate burden of disease (6). Taken together, these issues strongly suggest that additional, concerted efforts by multiple stakeholders (7, 8) are needed to tackle this severe early childhood disease.

Young children's health and care-seeking are largely determined by their family environment (9). Children's oral health care-related visits, oral health-related behaviors, attitudes, values, and habits are strongly influenced by their caregivers (10). For example, guardian's health literacy may determine their young children's optimal (i.e., preventive) vs. sub-optimal (i.e., problem-initiated) entry into the dental care system (11, 12), as well as future dental care-associated expenditures (13). Other studies have directly linked mothers' and children's dental caries status (14). Clearly, the road to improved children's oral health includes a focus on families and identifying means to empower parents and communities to better care for children's oral health (15). Adult family members shape the behavioral landscape underlying their young children's oral health; thus, it is logical to empirically study their specific roles, influencing factors, and areas for potential intervention.

Guardians' perceptions of their young children's oral health are useful proxies of their clinically determined dental needs (16–18) and crucially, they are known to influence dental care–seeking behaviors (11, 19, 20). Of note, guardian-reported child oral health was found to be the most informative element in one recent machine learning based ECC screening application (21). Recent reports examining parental perceptions of young children's oral health in diverse settings (22–26) demonstrate the value and practical utility of understanding the agreement between self-reports and actual clinical status or treatment needs, as well as factors influencing them. Studies among community samples (i.e., not actively dental care-seeking populations), preschool-age children (i.e., those whose oral health-related behaviors and care is entirely determined by their family environment), and diverse (i.e., multi-ethnic) populations are warranted.

The overarching motivation for this study was to add to the knowledge base of the association between guardians' reports of young children's oral health and children's clinically determined oral health status, examining factors influencing and potentially modifying these associations in a large, community-based sample of preschool-age children. This has not been previously done in a large, multi-ethnic, community-based sample of preschool-age children. Specifically, we sought to (1) examine the social and behavioral correlates of fair/poor guardian-reported child oral health, and (2) quantify the association of these reports with the prevalence of ECC, unrestored caries lesions and reported history of toothaches. We therefore hypothesize that an association exists between parental perception of a child's oral health and their clinically determined oral health status. Additionally, that those with fair/poor guardian-reported child oral health will have distinguishing characteristics spanning socio-demographics, oral-health related practices, diet, and presence of a dental home. Ultimately, if guardian reports prove valuable and informative for their children's oral health status, they may aid screening efforts to identify members of the population who are most in need of care.

## Methods

### Study Population

We used clinical and questionnaire data obtained in the ZOE 2.0 pediatric oral health study, a community-based cross-sectional epidemiologic study of childhood oral health in North Carolina (NC), United States (27, 28). Between 2016 and 2019, the investigators enrolled 8,059 children ages 36-71 months attending public preschools (Head Start) in 86 out of 100 NC counties. Head Start is a comprehensive program that provides education and healthcare services to low-income families, who are also eligible for public insurance. During the study period, there were ~20,000 children enrolled in the Head Start system in North Carolina; 13,089 children were invited to participate. Children's guardians provided written informed consent to participate in this IRB-approved study (UNC-Chapel Hill #14-1992) and completed a written questionnaire about their children's oral health. This questionnaire was available in both English and Spanish language. Comprehensive clinical examinations took place in children's preschool centers typically within 2 months of enrollment. In this study 8,059 3-5 year-old children were enrolled and all their parents/legal guardians returned the questionnaire. Furthermore, 6,470 (80%) had clinical examinations, and 6,328 of those yielded usable clinical and questionnaire data. Detailed information about the study population, sample size considerations, procedures, and the clinical examination protocol have been previously reported (27, 29).

### Measures and Variables

The questionnaire for the guardians included 15 items covering 5 domains of information: socio-demographics (i.e., gender, race/ethnicity, parents' level of education), oral health-related practices (i.e., frequency of brushing, use of fluoridated toothpaste, adult involvement in tooth brushing), diet (e.g., daily frequency of sugar-containing snacks and beverages), presence of a dental home, and guardian-reported child oral health status including proxy-reported health and history of toothaches (not due to teething). As defined by the American Academy of Pediatric Dentistry (AAPD), dental home is understood as a continuous relationship between a dentist and patient. As described here, having a dental home has proven to provide better health outcomes for children, especially those at higher risk for ECC or periodontal disease. To measure proxy-reported oral health, we used an item routinely used in the U.S. National Health and Nutrition Examination Survey (NHANES)—“how would you describe the condition of your child's mouth and teeth,” that included five response options: excellent, very good, good, fair, and poor. In the present study we dichotomized item responses to distinguish between “negative” (i.e., fair/poor) and “positive” (i.e., good/very good/excellent) reports. Ninety-nine percent (*n* = 7,965) of participants answered this question and this group comprised the study's analytical sample. Answers to the question regarding history of toothaches not due to teething were also treated as a dichotomous response variable. In addition to individual questionnaire item responses on oral health behaviors–OHB (i.e., diet/feeding practices, oral hygiene practices and presence of a dental home), we used a latent class analysis-derived membership variable “favorable vs. unfavorable OHB” that broadly segregates individual participants with oral health-promoting vs. deleterious oral health behaviors (30).

Clinical examinations were done by trained and calibrated dental examiners using modified visual International Caries Detection and Classification (ICDAS) criteria (31). Dental caries experience was recorded at the tooth surface-level and in this study was defined at the moderate/established caries lesion threshold (ICDAS ≥ 3) (32). Consequently, ECC cases were defined as children with decayed, missing, filled surface (dmfs) index ≥ 1 (i.e., at least one primary tooth surface with caries experience) and those with unrestored caries lesions had decayed surfaces index (ds) ≥ 1 (i.e., at least one caries-affected and not restored primary tooth surface). Of note, for the purposes of this study, we considered the presence of unrestored, ICDAS ≥ 3 caries lesions, to represent “unrestored disease.”

### Analytical Approach

We sought to determine the association of the dichotomized reports of oral health with the prevalence of ECC, unrestored caries lesions, and history of toothaches. We also examined correlates of fair/poor guardian-reported child oral health through responses derived from the questionnaire. For initial data description, we relied on descriptive and bivariate tabular and visual methods of presentation. There data were clustered in nature: specifically, children were enrolled in 260 different preschool centers (primary clusters) and within 34 different preschool programs (higher level clusters). We employed statistical methods to account for this study design feature. Bivariate comparisons (i.e., Pearson chi-square) and estimates of association [prevalence ratios (PR) and 95% confidence intervals (CI)] were obtained using Stata's *svy* function and Taylor-linearized variances. To obtain covariate-adjusted estimates of association of fair/poor oral health reports with clinical measures of disease (ECC and unrestored caries) and reported toothaches, we used multi-level generalized linear models (log-binomial models) including random-effect terms for the two-level nested clustered design and fixed-effect terms for children's age in months, race/ethnicity, and parents' education level. Interpretation of these model results was based on marginal effects estimation (33) and reporting of average marginal effects (A.M.E.) expressed in absolute percentage points (p.p.) increase in the prevalence of ECC, unrestored caries lesions, and toothaches. Analyses were done using Stata/MP version 17.0 (StataCorp LLC, Texas, US) and JMP Pro 16.0 (SAS Institute Inc., Cary, NC).

## Results

Participating children (*n* = 7,965) had mean age of 52 months and were of diverse racial/ethnic composition (Table 1), with 48% being non-Hispanic Blacks (African Americans), 20% Hispanic, and 18% non-Hispanic whites. The demographic composition of these participants remained the same for the subset with ECC clinical information (*n* = 6,328). The prevalence of fair/poor reported oral health in this sample of children was 15%. The frequency of fair/poor reports increased monotonically with children's age, was inversely associated with their guardians' educational attainment, and was higher among Hispanics (21%) and African Americans (15%) compared to their non-Hispanic white counterparts (11%). The associations of fair/poor reported child oral health with race/ethnicity and education persisted within and across strata of children's age.

**Table 1:** Sociodemographic information of the ZOE 2.0 study participants, overall and among those with clinical information for early childhood caries (ECC).

	All participants^*^	**w/ECC information**
	***n*** **(column %)**	***n*** **(column %)**
Entire sample	7,965 (100)	6,328 (100)
**Gender**		
Boy	3,955 (50)	3,155 (50)
Girl	4,008 (50)	3,173 (50)
**Age at enrollment (years)**		
3	2,537 (32)	1,967 (31)
4	4,183 (53)	3,334 (53)
5	1,245 (16)	1,027 (16)
(months), mean (SD)	52 (7.5)	52 (7.4)
**Race/ethnicity**		
Non-hispanic black	3,755 (48)	3,003 (48)
Hispanic	1,585 (20)	1,268 (20)
Non-hispanic white	1,422 (18)	1,122 (18)
>1 race	836 (11)	654 (10)
AI/AN/Asian/NH/PI/Other	285 (4)	219 (4)
**Guardian's education**		
Some elementary	367 (5)	317 (5)
Some high school	1,110 (14)	861 (14)
High school/GED diploma	2,941 (38)	2,338 (38)
Some technical/college education	2,243 (29)	1,783 (29)
College or more	1,118 (14)	885 (14)

* *94 participants (1.2% of the entire study population) were excluded from presentation and analysis due to missing information in the reported child oral health status questionnaire item*.

*ECC, early childhood caries; AI, American Indian; AN, Alaskan Native; NH, native Hawaiian, PI, Pacific Islander*.

Unfavorable patterns of child oral health-related behaviors were associated with higher prevalence of fair/poor reports (Table 2). Specific behaviors underlying this association included brushing less than twice a day, not having a dental home, and frequently consuming sugar-containing snacks (all with *P* < 0.0005). In contrast, we found no important associations with adult involvement in tooth brushing and use of a fluoride-containing toothpaste.

**Table 2:** Guardian-reported child oral health-related behavior information in the ZOE 2.0 study sample, overall and stratified by child reported oral health status.

		**Reported child oral health status**		
	All participants^*^	**Excellent/very good/good**	**Fair/poor**	
	***n*** **(column %)**	***n*** **(row %)**	***n*** **(row %)**	***P***
Entire sample	7,965 (100)	6,734 (85)	1,231 (15)	
**Pattern of modifiable child oral health-related behaviors** ^**†**^				<0.0005
Favorable	5,883 (74)	5,131 (87)	752 (13)	
Unfavorable	2,078 (26)	1,600 (77)	478 (23)	
**Tooth brushing frequency**				<0.0005
Twice a day or more	4,940 (62)	4,293 (87)	647 (13)	
Less than twice a day	3,011 (38)	2,431 (81)	580 (19)	
**Adult involvement in tooth brushing**				0.172
Yes	4,777 (60)	4,015 (84)	762 (16)	
No	3,180 (40)	2,711 (85)	469 (15)	
**Use of fluoridated toothpaste**				0.155
Yes	6,073 (77)	5,162 (85)	911 (15)	
No	848 (11)	713 (84)	135 (16)	
I do not know	980 (12)	804 (82)	176 (18)	
**Child has a dental home**				<0.0005
Yes	6,544 (84)	5,699 (87)	845 (13)	
No	1,267 (16)	907 (72)	360 (28)	
**Between-meal sugar-containing snacks and beverages daily consumption**				<0.0005
≥2	5,713 (72)	4,757 (83)	956 (17)	
<2	2,235 (28)	1,962 (88)	273 (12)	

* *94 participants (1.2% of the entire study population) were excluded from presentation and analysis due to missing information in the reported child oral health status questionnaire item*.

† *Derived from latent class analysis of responses to 6 modifiable oral health behavior questionnaire items, as reported by Simancas-Pallares et al. (30)*.

In bivariate comparisons, we found that children with fair/poor reported oral health had worse clinically determined oral health (Table 3). For example, they were twice as likely to have unrestored disease [prevalence ratio (PR) = 2.0; 95% confidence interval (CI) = 1.8-2.1] and 3.5 times as likely to have experienced toothaches [PR = 3.5; 95% CI = 3.1-3.9] compared to those with better reported oral health. After adjusting for children's age and race/ethnicity and guardian's education in multivariate analyses, these associations remained statistically significant and were of substantial magnitude. Sub-optimal (i.e., fair/poor) reported oral health was associated with absolute percentage point (p.p.) increases in ECC: +44 p.p., unrestored caries lesions: +26 p.p., and history of tooth aches: +15 p.p.

**Table 3:** Clinical and guardian-reported measures of child oral health in the ZOE 2.0 study sample, and associations with reported child oral health status.

	All participants^*^	**Child oral health status**			
		**Excellent/very good/good**	**Fair/poor**		
	***n*** **(column %)**	***n*** **(row %)**	***n*** **(row %)**	***PR*** **(95*%CI*)** ^‡^	***adjusted A.M.E**.^**||**^*
**ECC status** ^**†**^					
Yes	3,407 (54)	2,571 (75)	836 (25)	1.8 (1.7-1.9)	+42 p.p. (37-47)
No	2,921 (46)	2,797 (96)	124 (4)	ref.	ref.
**Unrestored disease**^**†**^ **status**					
Yes	2,269 (36)	1,682 (74)	587 (26)	2.0 (1.8-2.1)	+26 p.p. (21-30)
No	4,059 (64)	3,686 (91)	373 (9)	ref.	ref.
**History of tooth aches, not from teething**					
Yes	958 (12)	586 (61)	372 (39)	3.5 (3.1-3.9)	+15 p.p. (14-17)
No/I do not know	6,930 (88)	6,082 (88)	848 (12)	ref.	ref.

* *Participants with clinical information only are included in ECC and unrestored disease comparisons, whereas all participants with non-missing questionnaire information are included in the toothache history comparison*.

† *Defined at the ICDAS≥3 caries lesion detection threshold*.

‡ *Prevalence ratio and 95% confidence intervals estimated with an unadjusted log-binomial model accounting for the complex study design using Stata's svy function*.

|| *Adjusted average marginal effect (expressed in percentage points) of the association of reported child oral health status, with ECC, unrestored disease and history of toothaches, estimated from an adjusted multilevel generalized linear model including terms for features of study design, as well as children's age, race/ethnicity, and guardian's education*.

*ECC, early childhood caries; PR, prevalence ratio; CI, confidence interval; A.M.E., average marginal effect*.

## Discussion

This study sought to quantify the association between parental perception of children's oral health and their clinically determined dental needs. The findings provide support for the use of proxy reports, specifically guardian-provided assessments in investigations and monitoring of early childhood oral health at the population level. Child oral health status reports based on a single questionnaire item were found to be strongly indicative of clinically determined measures of ECC, including unrestored caries lesions and history of toothaches. These associations were robust to adjustments for children's and parents' sociodemographic characteristics. Importantly, these findings were generated from a large, community-based sample of preschool-age children, and not a clinic-ascertained convenience sample that might overrepresent a dental care-seeking subset of this population. Taken together, these data affirm the value of proxy-reported measures of child oral health and their concordance with clinical and public health-important oral disease endpoints.

We also sought to explore correlates of suboptimal (fair/poor) guardian-reported child oral health. Indeed, reports of sub-optimal (i.e., fair/poor) child oral health were significantly more prevalent among population subgroups known to experience disproportionate levels of dental disease—ethnic minorities and children in families with low levels of education. This is consistent with previous findings and may be due to dental care seeking attitudes (e.g., inconsistent dental care) or values (e.g., perception of primary teeth not being important). This association has emerged in both preschool-age and school-age populations. For example, Talekar et al. analyzed national data in the United States found worse reported oral health among preschool-age children whose parents had lower educational attainment (16). A more recent study (34) confirmed that lower parental education was associated with higher rates of decay in their children.

Strikingly, sub-optimal reports were almost twice as common among Hispanic participants compared to their non-Hispanic white counterparts, mirroring earlier reports of oral health disparities experienced by this population group (35, 36). This finding must be interpreted with caution, as Spanish speakers may differentially report child oral health problems—in an earlier study among a younger (6-23-month-old) child population in NC we found a lower rate of ‘child oral health-related problems' among children in Spanish-speaking families compared to their English-speaking counterparts (37). Low health literacy (24) and social desirability bias (38), among other reasons, could diminish the validity of guardians' child oral health reports. Nevertheless, this study's findings are concordant with previous reports suggesting considerable association between guardian reports and objective measures of childhood dental disease (16–18, 23, 39).

The monotonic increase of fair/poor reported child oral health status with children's age is expected, as ECC experience, severity, and associated problems also increase in the same manner at the population-level. It is possible that parents perceive visual changes in their children's teeth (i.e., the formation of cavities), signs and symptoms of tooth pain or sensitivity, or they are informed by a health professional that dental problems exist. The associations with sub-optimal child oral health-related behaviors and practices, such as infrequent brushing, lack of a dental home, and frequent consumption of sugar-containing snacks and beverages are demonstrative of the important role these, arguably modifiable, behavioral risk factors play in the development of ECC at the person-level (40). Emphasis is currently placed by multiple stakeholders on all children establishing a dental home in the first 12 months of life (41). Introduction of a dental home is believed to provide better health care outcomes for children, especially those at higher risk of developing ECC. Children introduced to a dental home at an early age receive effective preventative care (42). These children are also less likely to need emergency dental care, which is emotionally and financially burdensome (43, 44).

From a public health standpoint, the estimated associations of 44 percentage points higher prevalence of ECC, 26 percentage points higher prevalence of unrestored disease, and 15 percentage points higher prevalence of history of toothaches, are noteworthy—it would be justifiable to use population-wide screening strategies using single-item guardians' reports to identify segments of the child population that might need targeted or intensified comprehensive dental care. This may be especially important for largely non-dental care-seeking segments of the population wherein clinical information is lacking, or where access to dental care services is difficult. This strategy may also offer cost and time advantages and reduce study participants' information burden.

The study's findings must be viewed while acknowledging several limitations. First and foremost, questionnaire and clinical data were obtained practically contemporaneously—this limits any potential for causal inference (i.e., guardians' reports being predictive of clinical disease or toothaches), but these identified associations can be validly interpreted in the context of screening. Second, it is possible that some guardians' perceptions were influenced by dental care received by their children—even if presenting with no current clinical problems, guardians' perceptions of oral health may be lowered due to the history of recent and potentially extensive dental care. Notwithstanding this speculative scenario, the group of children with ECC experience include both those with restored and unrestored disease and identifying both groups is of public health significance, i.e., for monitoring disease experience. Finally, these results emanate from a single state in the U.S., and from a low-income, high-risk child population attending public preschools; while this population may not be representative of the general preschool-age population in other U.S. states or countries, or those in more affluent strata, we believe that the findings regarding guardians' reports are generally transferable across samples and populations.

## Conclusions

In this cross-sectional study among a multi-ethnic, community-based sample of preschool-age children, we found strong associations between guardian-reported child oral health status, and ECC, including unrestored caries lesions, and history of toothaches. Fair/poor child oral health reports were associated with lower guardian education and were higher among racial/ethnic minorities compared to non-Hispanic whites. We conclude that guardian reports of their children's oral health are valuable indicators of clinical and public health-important child oral disease endpoints.

## Data Availability Statement

The datasets analyzed in this study can be found online in the Carolina Digital Repository as “ZOE 2.0: A community-based, epidemiologic study of early childhood oral health” at: https://doi.org/10.17615/8yjy-w790 (accessed 1 August 2021).

## Ethics Statement

The study was reviewed and approved by the Institutional Review Board of the University of North Carolina-Chapel Hill. Written informed consent to participate in this study was provided by the participants' legal guardian/next of kin.

## Author Contributions

EI, JG, PS, MS-P, and KD made substantial contributions to the conceptualization of the work, data collection, data analysis, co-wrote sections of the paper, read, and approved the final version of the manuscript. All authors have agreed both to be personally accountable for each author's own contributions and to ensure that questions related to the accuracy or integrity of any part of the work are appropriately investigated.

## Funding

This study was funded by a grant from the National Institutes of Health/National Institute of Dental and Craniofacial Research U01-DE025046 (PI: KD). The funders had no role in the design of the study; in the collection, analyses, or interpretation of data; in the writing of the manuscript, or in the decision to publish the results.

## Conflict of Interest

The authors declare that the research was conducted in the absence of any commercial or financial relationships that could be construed as a potential conflict of interest.

## Publisher's Note

All claims expressed in this article are solely those of the authors and do not necessarily represent those of their affiliated organizations, or those of the publisher, the editors and the reviewers. Any product that may be evaluated in this article, or claim that may be made by its manufacturer, is not guaranteed or endorsed by the publisher.

## Abbreviations

ECC: early childhood caries
ICDAS: International Caries Detection and Assessment System
dmfs: the number of decayed, missing, and filled primary tooth surfaces due to caries
ds: the number of decayed primary tooth surfaces
PR: prevalence ratio
CI: confidence interval
A.M.E.: average marginal effect.
